# Suspected atrioesophageal fistula complicated by coronary air embolism after catheter ablation for atrial fibrillation: direct angiographic visualization and catheter aspiration in a case report

**DOI:** 10.3389/fcvm.2026.1985470

**Published:** 2026-10-15

**Authors:** Chao Yang, Xiaobin Zeng, Shihai Wang, Chaoju Wang, Jianjun Lan, Yahong Wen, Zaiyong Zheng

**Affiliations:** Department of Cardiology, Panzhihua Central Hospital, Sichuan, China

**Keywords:** atrial fibrillation, atrioesophageal fistula, case report, catheter ablation, coronary air embolism

## Abstract

Atrioesophageal fistula is a rare but fatal complication of catheter ablation for atrial fibrillation(AF). It may present with infection, dysphagia or other swallowing-related symptoms, neurologic events, and systemic air embolism; coronary air embolism is even less common.

**Case summary:**

An 84-year-old woman was admitted with recurrent palpitations lasting 1 h, approximately 1 month after radiofrequency catheter ablation for atrial fibrillation and left atrial appendage closure(RFCA & LAAC). On admission, she was in sinus rhythm and had a normal cardiac troponin level. Four hours after admission, she abruptly became unresponsive and developed fever and severe respiratory abnormalities. Electrocardiography subsequently showed ST-segment elevation in leads II, III, and aVF. Emergency coronary angiography demonstrated gas in the mid right coronary artery with TIMI 0 distal flow, and catheter aspiration was performed. Computed tomography (CT) of the head and chest revealed gas at multiple sites, including the intracranial compartment, cardiac chambers, and great vessels. Blood cultures grew Streptococcus constellatus and other streptococci. Given the recent left atrial ablation, acute neurologic and myocardial ischemic manifestations, multisite intravascular gas, and bacteremia with organisms associated with the oropharyngeal or gastrointestinal flora, an atrioesophageal fistula complicated by systemic air embolism was strongly suspected. Her condition deteriorated rapidly, and she developed cardiac arrest and died approximately 27 h after admission.

**Conclusion:**

Atrioesophageal fistula and coronary air embolism should be considered in patients who develop fever, impaired consciousness, or acute ST-segment elevation within several weeks of atrial fibrillation ablation. Coronary angiography can directly visualize intracoronary gas and allow catheter aspiration; however, the certainty of an atrioesophageal fistula diagnosis should remain commensurate with the available imaging evidence.

## 1 Introduction

Atrioesophageal fistula (AEF) is a rare but catastrophic complication of catheter ablation for AF and typically develops several weeks after the procedure (1, 2). Because early symptoms are nonspecific, patients may initially present with fever, chest pain, swallowing discomfort, altered consciousness, seizures, or focal neurologic deficits (3). Delayed diagnosis often leads to severe infection, systemic air embolism, and death (3, 4). Coronary air embolism is an even rarer initial or predominant cardiac manifestation. We report on a patient who developed right coronary air embolism with intracranial and multisite intravascular gas approximately 1 month after RFCA & LAAC (Figure 1).

**Figure 1:**
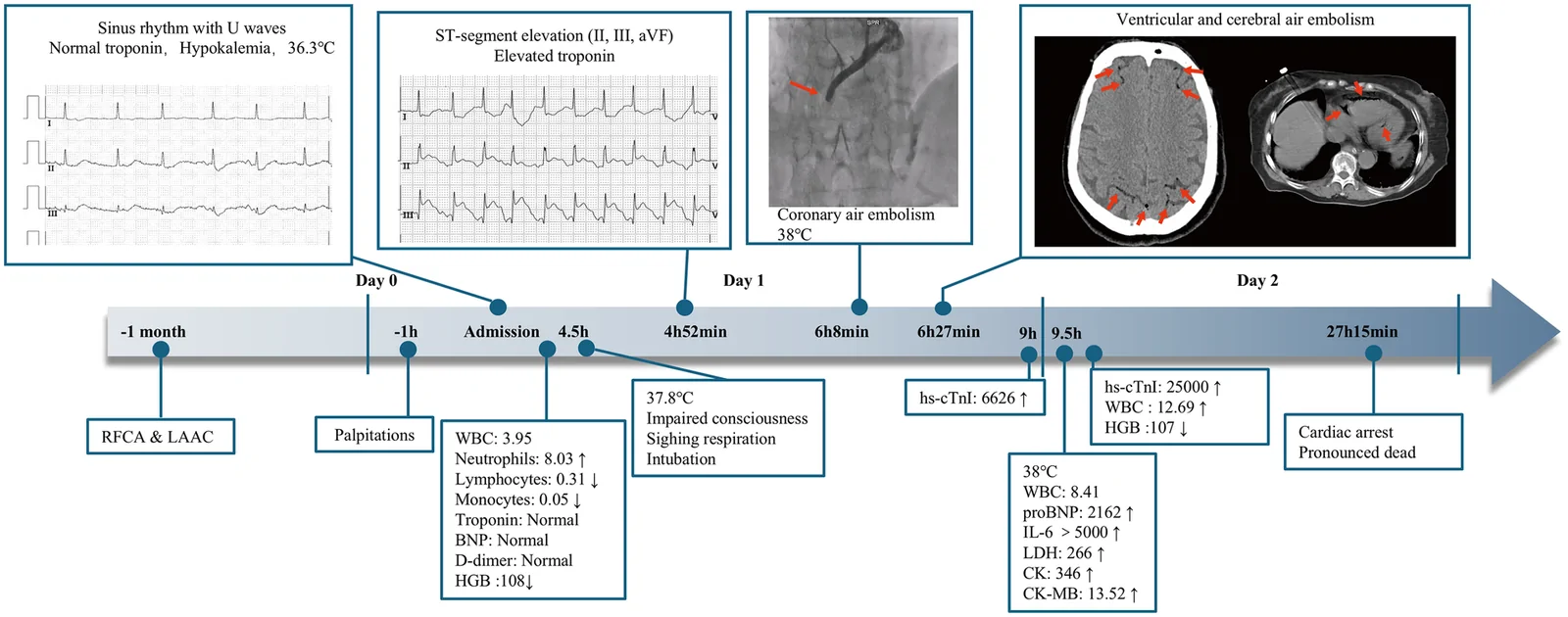
Timeline of the patient's clinical course from hospital admission to death. Time 0 denotes the time of hospital admission. All temperatures shown represent the patient's body temperature. Units for the laboratory measurements are provided in the main text. Point-of-care testing reference ranges: myoglobin <108.6 ng/mL; cardiac troponin I (cTnI) < 0.1 ng/mL; creatine kinase–myocardial band (CK-MB) < 3.741 ng/mL. Laboratory reference ranges: high-sensitivity cardiac troponin I(hs-cTnI) < 28.64 ng/L; pro-B type natriuretic peptide (pro-BNP) < 1,800 pg/mL; interleukin-6 (IL-6) < 7 pg/mL; lactate dehydrogenase (LDH) 120-250 U/L; creatine kinase (CK) 40-310 U/L; CK-MB <5.0 ng/mL.

## 2 Case presentation

### 2.1 Medical history and presentation

An 84-year-old woman with a history of hypothyroidism had undergone a combined radiofrequency catheter ablation for atrial fibrillation and left atrial appendage closure at another hospital approximately 1 month earlier; procedural details were unavailable. While visiting Panzhihua, which involved an increase in altitude of approximately 600 m, she was admitted because of recurrent palpitations lasting 1 h. On admission (time 0), she was fully conscious and denied fever, chest pain, chills, or other symptoms. Her heart rate was 82 beats/min, respiratory rate 20 breaths/min, blood pressure 116/57 mmHg, and peripheral oxygen saturation (SpO₂) was 90%. Electrocardiography showed sinus rhythm and T-wave inversion in leads V2-V6. High-sensitivity cardiac troponin I, B-type natriuretic peptide（BNP）, and D-dimer levels were not elevated. Her serum potassium level was 2.34 mmol/L. A complete blood count showed a white blood cell (WBC) count of 3.95 × 10⁹/L, neutrophil count of 3.53 × 10⁹/L, lymphocyte count of 0.35 × 10⁹/L, hemoglobin level of 108 g/L, and platelet count of 158 × 10⁹/L. The potassium level normalized after supplementation.

### 2.2 Acute deterioration, coronary angiography, and imaging

At 4.5 h after admission, the patient abruptly became unresponsive. Her temperature was 37.8 °C, and she developed gasping respirations, prompting emergency endotracheal intubation and resuscitative measures. At 4 h and 52 min, repeat electrocardiography showed ST-segment elevation in leads II, III, and aVF (Figure 2). Point-of-care testing showed a myoglobin concentration of 225.10 ng/mL, cardiac troponin I of 0.111 ng/mL, and CK-MB of 0.4196 ng/mL. Emergency coronary angiography was performed approximately 6 h after admission. It showed approximately 50% stenosis in the mid left anterior descending artery. Gas was already visible in the mid right coronary artery during the first contrast injection, with TIMI 0 distal flow. Catheter aspiration removed a large volume of gas without thrombus, after which antegrade flow recovered to TIMI grade 3. The patient did not regain consciousness during the procedure.

**Figure 2:**
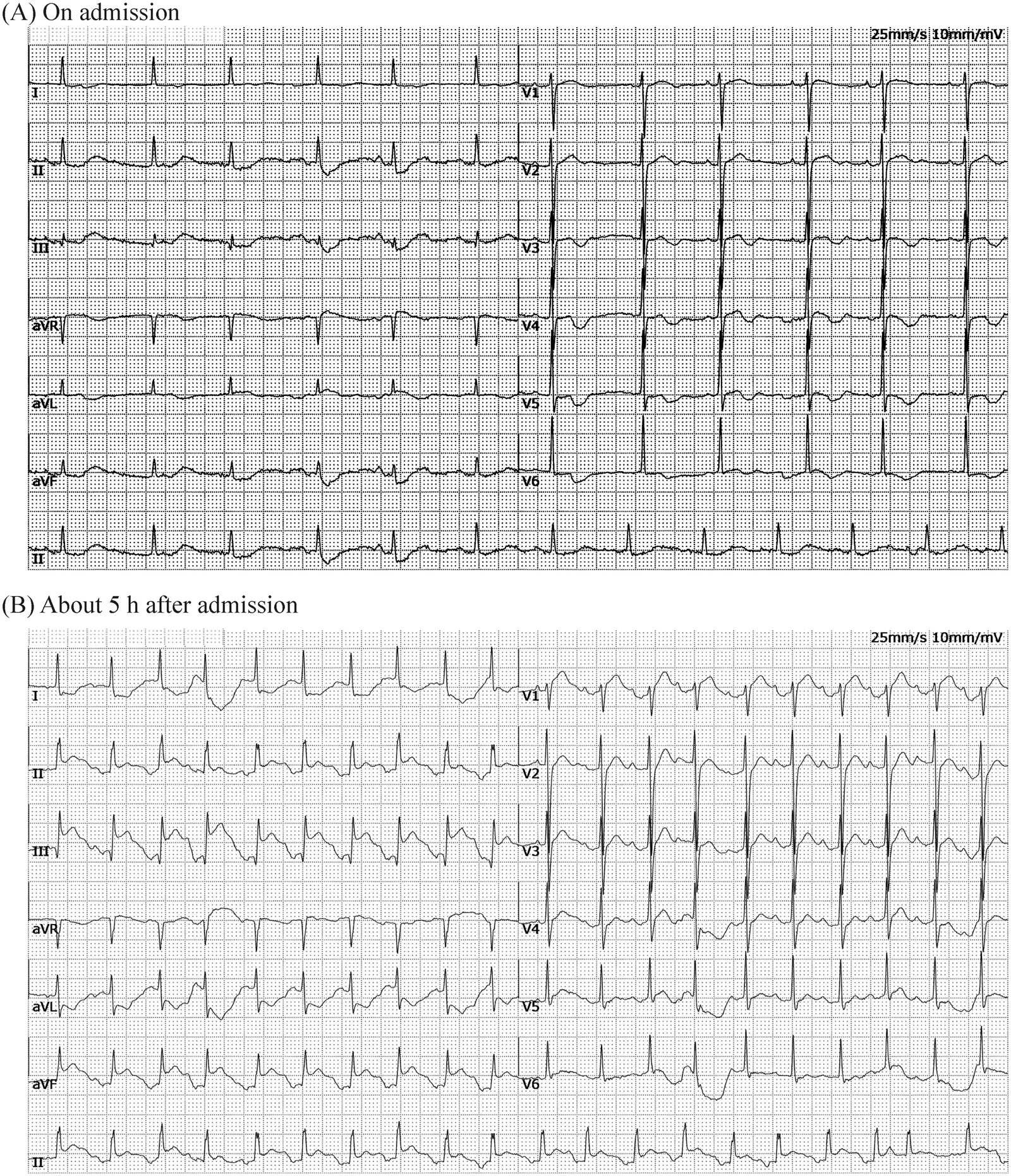
Electrocardiograms obtained on admission and 5 h after admission. **(A)** On admission, sinus rhythm with T-wave inversion in leads V2–V6. **(B)** Five hours after admission, ST-segment elevation in leads II, III, and aVF.

Non-contrast computed tomography (CT) of the head and chest was completed approximately 6.5 h after admission. Head CT showed multiple gas-density foci along the bilateral cortical sulci and in the region of the superior sagittal sinus. Chest CT showed gas-density foci in all cardiac chambers and in the regions of the aorta, coronary arteries, pulmonary arteries, and cervical vessels (Figures 3, 4). Retrospective image review identified a suspected tract between the posterior wall of the left atrium and the adjacent esophagus (Figure 5). Bedside echocardiography showed a left ventricular ejection fraction of 47%, a small left-to-right shunt through the mid interatrial septum measuring approximately 2.5 mm in width, and a minimal pericardial effusion with a maximal depth of approximately 8 mm. Contrast-enhanced CT was not performed because of the patient's critical condition and hemodynamic instability. Upper gastrointestinal endoscopy and transesophageal echocardiography were also avoided because of the risk of introducing additional air.

**Figure 3:**
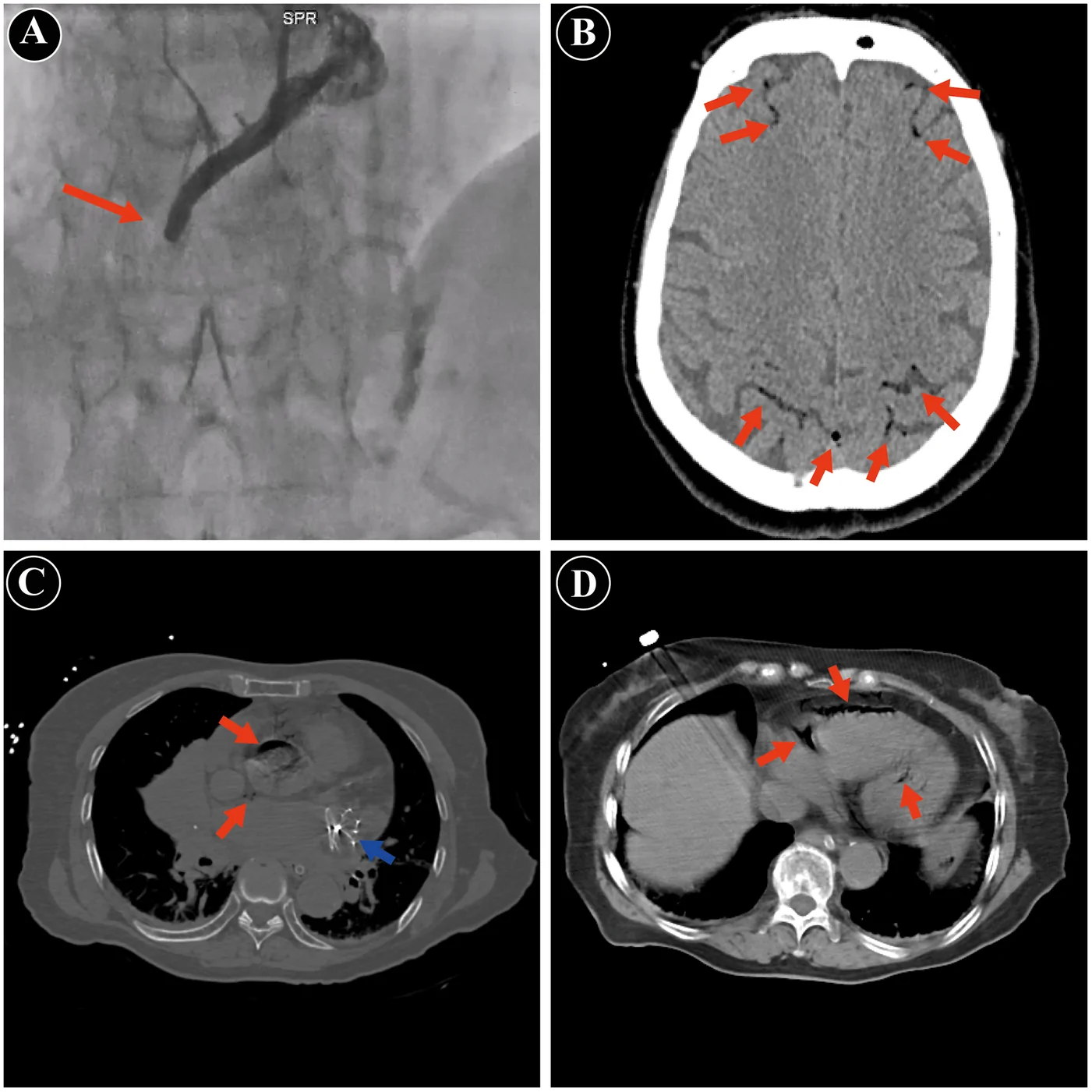
Multimodal findings of suspected atrioesophageal fistula with systemic air embolism. **(A)** Coronary angiography demonstrating air embolism in the mid right coronary artery.**(B)** Brain CT showing multiple intracranial air densities.**(C)** Chest CT showing extensive air within the aorta (upper red arrow) and coronary arteries (lower red arrow); the previously implanted left atrial appendage occluder is also visible (blue arrow).**(D)** Chest CT revealing extensive air within aorta, right ventricle, and left ventricle(red arrows).

**Figure 4:**
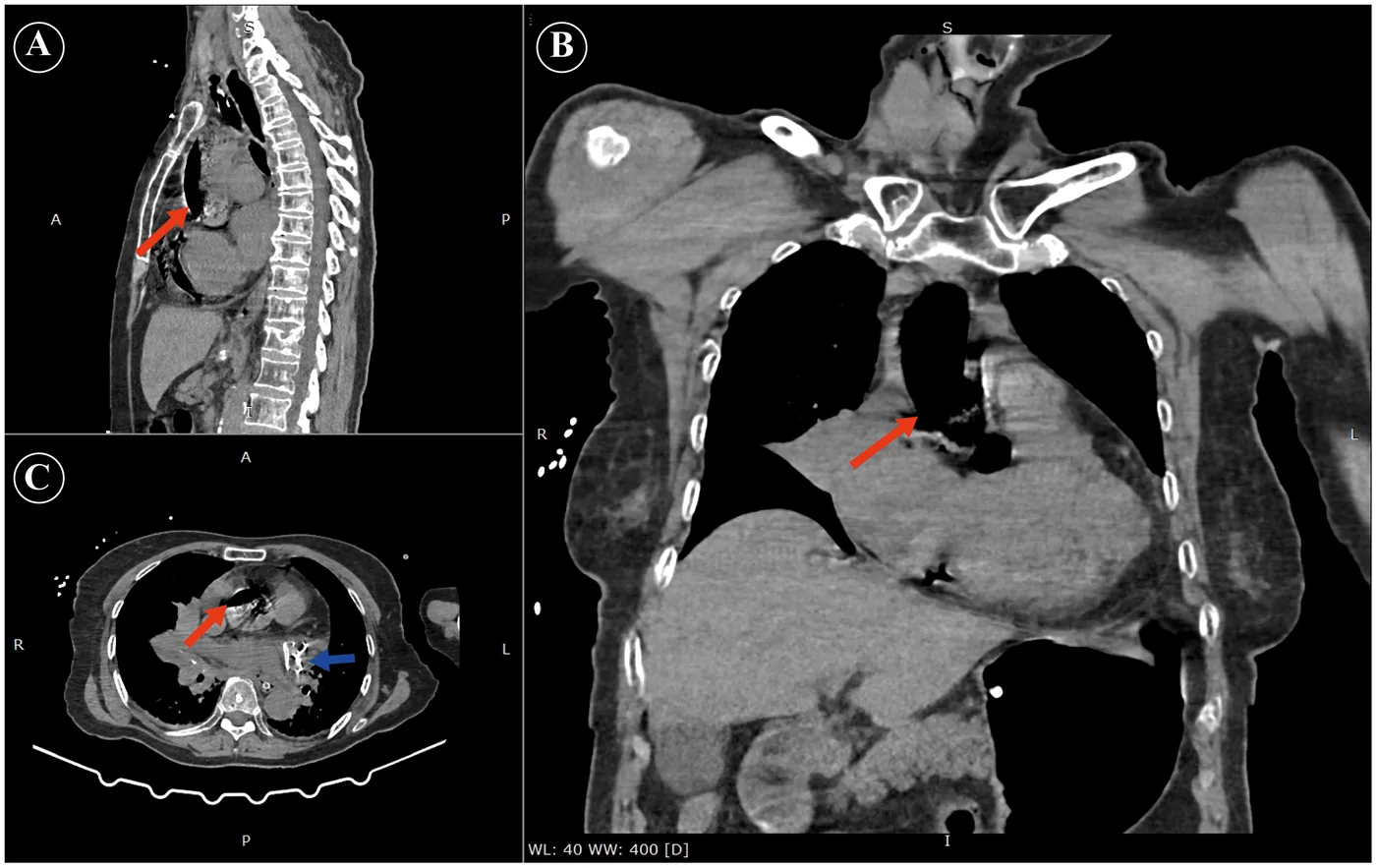
Multiplanar CT reformations show aortic air embolism in the sagittal **(A)**, coronal **(B)**, and axial **(C)** planes. and left atrial appendage occluder (blue arrow).

**Figure 5:**
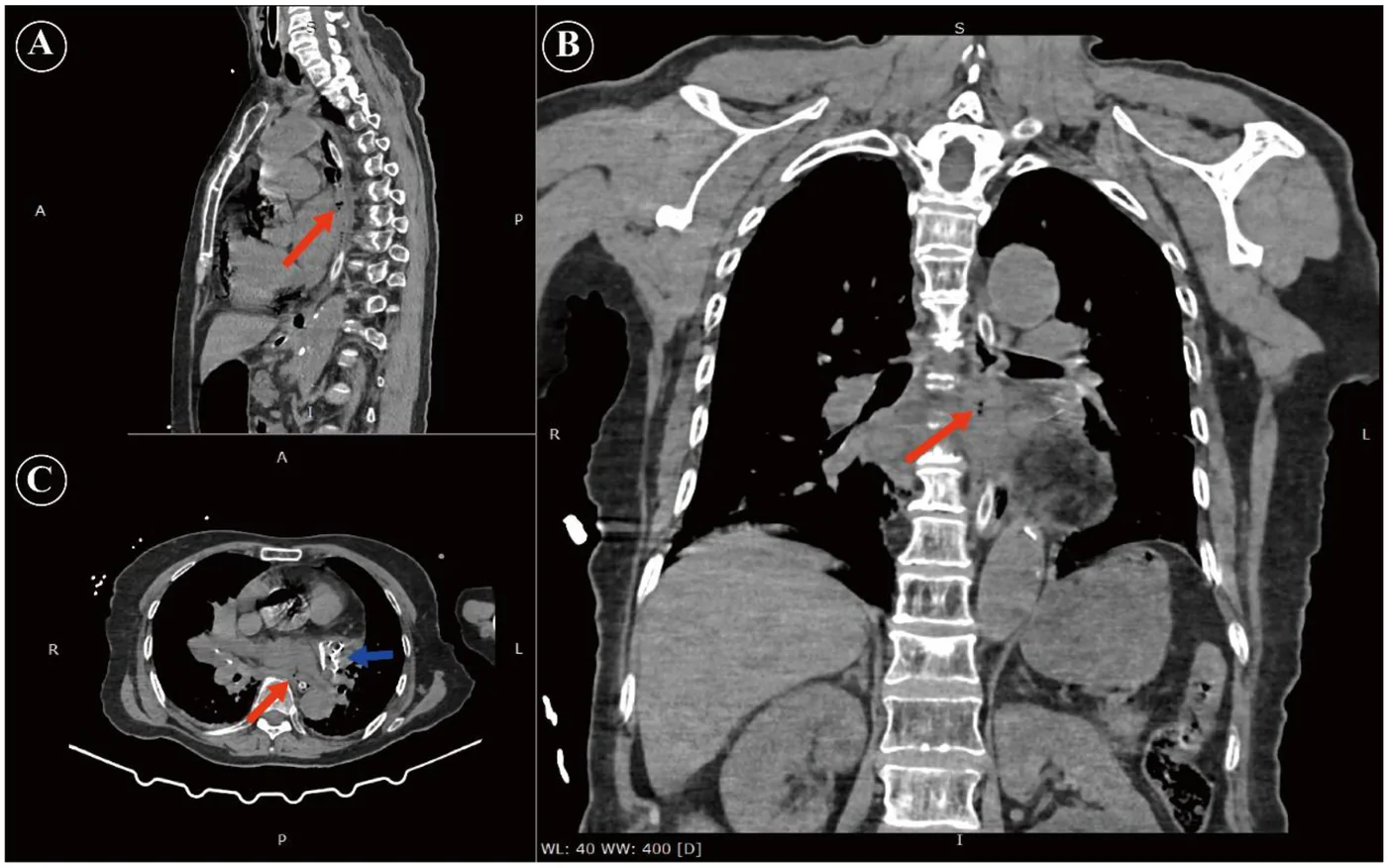
Multiplanar CT reformatted images suggest a possible fistulous tract between the left atrium and the esophagus (red arrow); the previously implanted left atrial appendage occluder is also visible (blue arrow).

### 2.3 Laboratory findings, treatment, and outcome

The patient received aspirin, atorvastatin, enoxaparin, piperacillin-tazobactam, and supportive care. Laboratory testing 10 h after admission showed a hs-cTnI level of 6,626 ng/L, alpha-hydroxybutyrate dehydrogenase of 216 U/L, LDH of 266 U/L, CK of 346 U/L, CK-MB of 13.52 ng/mL, pro-BNP of 2,162 pg/mL, andIL-6 of >5,000 pg/mL. The hs-cTnI level subsequently increased to >25,000 ng/L. Blood cultures grew Streptococcus constellatus and a small quantity of other streptococci. Surgical repair was deferred due to refractory shock requiring vasopressor support. Recurrent bradycardia and hypotension occurred during the subsequent course, and the patient developed cardiac arrest and died approximately 27 h and 15 min after admission.

## 3 Discussion

Radiofrequency energy was the first energy source used for atrial fibrillation ablation and remains the most widely used. It disrupts electrical connections between the pulmonary veins and the left atrium by producing coagulative necrosis through high temperatures (5). However, because thermal energy is not tissue selective, adjacent structures, including the esophagus and aorta, may also sustain thermal injury (6). In one study, endoscopic examination after RF ablation for atrial fibrillation in 267 patients identified esophageal ulcers in 2.2% of patients. The study also found that the distance between the esophagus and the left atrium was an important risk factor (7). Esophageal ulceration appears to precede fistula formation, which may partly explain why atrioesophageal fistula typically develops 19.3 ± 12.6 days after ablation (8). Pre-existing or post-ablation gastroesophageal reflux may contribute to the progression of an esophageal ulcer to a fistula (9). Active esophageal cooling devices may reduce the risk of atrioesophageal fistula (10).

An atrioesophageal fistula is direct communication between the left atrium and the esophagus. It is a rare but potentially fatal complication of RF ablation for atrial fibrillation (11). Demonstration of a fistulous tract between the esophagus and the left atrium on contrast-enhanced CT or magnetic resonance imaging, or direct visualization of the fistula, constitutes direct diagnostic evidence. However, most investigators agree that esophageal endoscopy should be avoided when an atrioesophageal fistula is suspected, as insufflation may force gas and esophageal contents through the fistula into the left atrium (8).

Once a fistula has formed, gas, fluid, and commensal microorganisms from the esophagus can enter the left atrium and subsequently reach the systemic circulation, resulting in air embolism and bloodstream infection (12, 13). Accordingly, oral or esophageal commensal organisms were isolated from blood cultures in our patient, as in several previously reported cases (11, 14, 15).

No standardized clinical diagnostic criteria for AEF have been established (16). A recent history of atrial fibrillation ablation is an important diagnostic clue. Contrast-enhanced chest CT is the preferred initial imaging modality (15). Potential findings include air within the left atrium or mediastinum, esophageal wall thickening, pericardial effusion, mediastinal inflammatory changes, and a suspected fistulous tract. However, a negative initial CT scan does not exclude AEF (17). Brain imaging may reveal cerebral infarction or cerebral air embolism. In patients with suspected bloodstream infection, isolation of oral or upper gastrointestinal commensals from blood cultures may provide an additional diagnostic clue (18).

### 3.1 Evidence supporting the diagnosis of atrioesophageal fistula

This case occurred approximately 1 month after radiofrequency ablation of the left atrium, a time course consistent with the typical delayed presentation of AEF (19). The patient presented with palpitations and initially had no biomarker or electrocardiographic evidence of acute myocardial injury. She then rapidly developed fever, altered consciousness, inferior ST-segment elevation, gas in the right coronary artery, intracranial and systemic intravascular gas, and Streptococcus constellatus bacteremia. Together, these findings raised a strong clinical suspicion of AEF, particularly the combination of recent ablation at the posterior left atrial wall, a neurologic event, systemic air embolism, and bacteremia with organisms associated with the oropharyngeal or gastrointestinal flora. The altered consciousness and ST-segment elevation preceded coronary angiography, and gas was clearly present during the first right coronary injection, supporting the conclusion that coronary air embolism preceded the angiographic procedure. However, because the patient's condition was critical and deteriorated rapidly, direct confirmation by contrast-enhanced CT, surgery, or autopsy was not obtained.

### 3.2 Possible mechanisms of coronary air embolism and gas in the right heart

Once an AEF has formed, intraluminal esophageal gas may enter the left atrium during swallowing, coughing, or changes in esophageal pressure. It can then pass into the systemic circulation and cause air embolism in the brain, coronary arteries, or other organs. In this patient, inferior ST-segment elevation was anatomically and temporally consistent with TIMI grade 0 flow in the right coronary artery. Angiography also documented interruption of the contrast column by gas, and catheter aspiration provided a direct means of restoring coronary perfusion.

Communication between the esophagus and left atrium most directly explains gas in the left heart and systemic circulation. The interatrial shunt may account for gas in the right ventricle, pulmonary artery, and other right-sided structures. However, because CT was performed after intubation, resuscitation, and coronary angiography, these interventions may also have altered the distribution of gas.

### 3.3 Management and clinical implications

Once AEF is confirmed or strongly suspected, urgent multidisciplinary evaluation involving both cardiac and thoracic surgeons is warranted, and definitive surgical treatment should not be delayed. Circulatory and respiratory support should be provided, broad-spectrum antimicrobial therapy covering oropharyngeal and gastrointestinal flora should be started promptly, and the feasibility of definitive surgical repair should be assessed. Published pooled analyses indicate that both endoscopic and surgical treatment are associated with substantially lower mortality than conservative management (3, 4, 8, 20, 21). The key surgical principles include debridement of nonviable tissue and repair of both the left atrial and esophageal defects. The left atrial defect may be closed primarily or repaired with a pericardial patch, whereas the esophageal defect may be closed in layers, preferably with interposition of a vascularized tissue flap between the repaired structures. Esophagectomy may be required when the esophageal injury is extensive or the remaining tissue is unsuitable for primary repair. Cardiopulmonary bypass should be considered, particularly in hemodynamically unstable patients or when adequate debridement and intracardiac repair cannot otherwise be achieved (17, 22). Because isolated esophageal stenting does not address the left atrial defect, it should generally not be regarded as definitive treatment for a true AEF. Nevertheless, it may be considered as a bridge to definitive surgery in selected patients when immediate operative repair is not feasible (23, 24). Emergency measures for coronary air embolism include high-concentration oxygen, maintenance of coronary perfusion, and, when appropriate, catheter aspiration.

Three features of this case merit emphasis. Neurologic deterioration and inferior ST-segment elevation occurred before coronary angiography. Angiography then directly demonstrated gas in the right coronary artery with TIMI 0 flow and enabled catheter aspiration. Finally, the combination of fever, altered consciousness, and acute ST-segment elevation several weeks after atrial fibrillation ablation should prompt immediate consideration of AEF and systemic air embolism in addition to acute coronary syndrome.

### 3.4 Limitations

This report has several limitations. First, contrast-enhanced CT did not directly demonstrate a fistulous tract, and the diagnosis of AEF was not confirmed by surgery or autopsy; it therefore rests primarily on the clinical presentation and indirect imaging evidence. Second, CT was performed after resuscitation, endotracheal intubation, and coronary intervention, so a procedural contribution to some of the intravascular gas cannot be completely excluded. Finally, the rapid clinical deterioration limited the completeness of the hemodynamic, neurologic, and laboratory data. These limitations require cautious interpretation of causal relationships.

## 4 Conclusion

Suspected AEF complicated by systemic and coronary air embolism should be considered early when fever, altered consciousness, and acute ST-segment elevation develop within several weeks of catheter ablation for atrial fibrillation. Coronary angiography can directly identify intracoronary gas and provide an opportunity for catheter aspiration. Rapid imaging assessment, multidisciplinary consultation, and evaluation for definitive surgical repair may be critical to improving outcomes.

## Data Availability

The original contributions presented in the study are included in the article/Supplementary Material, further inquiries can be directed to the corresponding author.

## Supplementary material

SUPPLEMENTARY VIDEO S1 Right coronary angiography shows abnormal contrast behavior. With antegrade injection, the contrast briefly moves forward and then washes back, consistent with intracoronary air embolism (red arrows). Several round, lucent areas are visible within the coronary lumen, compatible with intraluminal air.

SUPPLEMENTARY VIDEO S2 Coronary angiography demonstrates marked stagnation of contrast within the coronary artery.

SUPPLEMENTARY VIDEO S3 After intracoronary air aspiration, repeat angiography shows antegrade flow reaching the distal segment, although contrast transit remains slow.

SUPPLEMENTARY VIDEO S4 On repeat angiography shortly after air aspiration, antegrade contrast flow to the distal vessel improves further and appears near normal.

SUPPLEMENTARY VIDEO S5 Left main coronary angiography visualizes a previously implanted left atrial appendage occlusion device.
